# Luteolin Orchestrates Porcine Oocyte Meiotic Progression by Maintaining Organelle Dynamics Under Oxidative Stress

**DOI:** 10.3389/fcell.2021.689826

**Published:** 2021-06-15

**Authors:** Soo-Hyun Park, Pil-Soo Jeong, Ye Eun Joo, Hyo-Gu Kang, Min Ju Kim, Sanghoon Lee, Bong-Seok Song, Sun-Uk Kim, Seong-Keun Cho, Bo-Woong Sim

**Affiliations:** ^1^Futuristic Animal Resource and Research Center, Korea Research Institute of Bioscience and Biotechnology, Cheongju, South Korea; ^2^Department of Animal Science, College of Natural Resources and Life Science, Pusan National University, Miryang, South Korea; ^3^Department of Animal Science and Biotechnology, College of Agriculture and Life Science, Chungnam National University, Daejeon, South Korea; ^4^Department of Functional Genomics, University of Science and Technology, Daejeon, South Korea; ^5^Department of Animal Science, College of Natural Resources and Life Science, Life and Industry Convergence Research Institute, Pusan National University, Miryang, South Korea

**Keywords:** luteolin, antioxidant, oxidative stress, organelle dynamics, *in vitro* maturation, porcine oocyte

## Abstract

Increasing evidence has demonstrated that oxidative stress impairs oocyte maturation, but the underlying mechanisms remain largely unknown. Here, for the first time, we examined the antioxidant role of luteolin in meiotic progression and the underlying mechanisms. Supplementation of 5 μM luteolin increased the rates of first polar body extrusion and blastocyst formation after parthenogenetic activation, and the expression levels of oocyte competence (*BMP15* and *GDF9*)-, mitogen-activated protein kinase (*MOS*)-, and maturation promoting factor (*CDK1* and *Cyclin B*)-related genes were also improved. Luteolin supplementation decreased intracellular reactive oxygen species levels and increased the expression levels of oxidative stress-related genes (*SOD1*, *SOD2*, and *CAT*). Interestingly, luteolin alleviated defects in cell organelles, including actin filaments, the spindle, mitochondria, the endoplasmic reticulum, and cortical granules, caused by H_2_O_2_ exposure. Moreover, luteolin significantly improved the developmental competence of *in vitro*-fertilized embryos in terms of the cleavage rate, blastocyst formation rate, cell number, cellular survival rate, and gene expression and markedly restored the competencies decreased by H_2_O_2_ treatment. These findings revealed that luteolin supplementation during *in vitro* maturation improves porcine meiotic progression and subsequent embryonic development by protecting various organelle dynamics against oxidative stress, potentially increasing our understanding of the underlying mechanisms governing the relationship between oxidative stress and the meiotic events required for successful oocyte maturation.

## Introduction

Understanding the *in vitro* maturation (IVM) of oocytes is important for developing assisted reproductive technology (ART) and generating mature oocytes that are capable of successful embryonic development (Hashimoto, 2009). However, only a small percentage of immature oocytes can develop into blastocysts and subsequently result in pregnancy (Kwak et al., 2012). As low-quality oocytes resulting from improper IVM conditions are one of the main reasons for ART failure, optimization of IVM conditions is vital for improving ART (Lee et al., 2020).

Oocyte maturation involves both nuclear and cytoplasmic maturation. Nuclear maturation mainly refers to chromosome segregation and reflects the capacity of the oocyte to resume meiosis (Lopes et al., 2019). Cytoplasmic maturation involves the accumulation of mRNA, proteins, and substrates required for subsequent fertilization ability and developmental competence (Watson, 2007). Cell organelles including mitochondria, the endoplasmic reticulum (ER), and microtubules play important roles in both nuclear and cytoplasmic maturation through the regulation of protein and ATP synthesis and chromosome segregation (Mao et al., 2014). However, inadequate *in vitro* conditions lead to the abnormal behavior of cell organelles and finally result in disrupted meiotic maturation (Ueno et al., 2005; De los Reyes et al., 2011; De los Reyes et al., 2012).

In aerobic organisms, reactive oxygen species (ROS) including hydroxyl radicals, superoxide anions, and hydrogen peroxide (H_2_O_2_) are produced as byproducts of cell metabolism. However, during IVM cultivation of cumulus oocyte complexes (COCs), the IVM medium has fewer antioxidant enzymes than does the *in vivo* environment comprising follicular and oviduct fluids provided by the mother (Ye et al., 2017). The imbalance between ROS production and clearance caused by the lack of maternal antioxidants induces oxidative stress, which disrupts oocyte maturation and subsequent embryonic development (Khazaei and Nematbakhsh, 2004). Numerous studies have shown that antioxidant treatments such as adding quercetin, vitamin C, and resveratrol to IVM medium are helpful in improving oocyte quality (Sovernigo et al., 2017). Nevertheless, it is essential to elucidate the mechanisms underlying the relationship between oxidative stress and meiotic events for successful oocyte maturation.

Luteolin (3, 4, 5, 7-tetrahydroxyflavone; Lut) is a flavone, a type of flavonoid, usually found in broccoli, rosemary, olive oil, and peppermint. Accumulating studies have shown that Lut possesses anti-inflammatory, anticancer, and cytoprotective properties (Gupta et al., 2018). Especially, Lut functions as an antioxidant, protecting various cell types against oxidative stress (Xia et al., 2014; An et al., 2016). However, no studies have examined the effects of Lut on mammalian oocytes. In the present study, we investigated the role of Lut on meiotic progression using porcine oocyte because of the physiological and phylogenetic similarities between porcine and human oocyte, such as oocyte diameter and time period of maturation, which means porcine oocyte can be used to reflect the reproduction system of human (Day, 2000; Santos et al., 2014; Wang et al., 2016). In the present study, we explored the antioxidant effect of Lut supplementation on oocyte maturation and the subsequent developmental competence of *in vitro* fertilization (IVF) embryos. Given the important role of Lut in oocyte maturation, we also examined the role oxidative stress plays in various changes in cell organelles during oocyte maturation.

## Materials and Methods

### Chemicals

All chemicals and reagents were purchased from Sigma-Aldrich Chemical Co. (St. Louis, MO, United States) unless otherwise indicated.

### IVM

Porcine ovaries obtained from a local slaughterhouse were transported in 0.9% saline supplemented with 75 μg/mL potassium penicillin G and 50 μg/mL streptomycin sulfate and maintained at 37–38°C. COCs were aspirated from follicles (3–6 mm in diameter) through a disposable 10-mL syringe with an 18-gauge needle. The COCs were washed in 0.9% saline containing 0.1% bovine serum albumin (BSA), and 40–50 oocytes were matured in 500 μL of IVM medium in a four-well multi-dish (Nunc, Roskilde, Denmark) at 38.5°C under 5% CO_2_. For the first 22 h, Tissue Culture Medium 199 supplemented with 10% porcine follicular fluid, 0.57 mM cysteine, 10 ng/mL epidermal growth factor, 10 IU/mL pregnant mare serum gonadotropin (PMSG), and 10 IU/mL human chorionic gonadotropin (hCG) was used for maturation. During the second stage (22–44 h), the same medium was used without PMSG and hCG. After IVM, the cumulus cells were removed by repeated pipetting in 0.1% hyaluronidase. Denuded oocytes were classified as immature, degenerate, or at metaphase II (MII; first polar body extrusion visible) under a microscope (Nikon, Tokyo, Japan), and only MII oocytes were used for further experiments.

### Chemical Treatment

A stock solution of 20 mM Lut was prepared with dimethylsulfoxide and diluted in IVM medium to final concentrations of 0 (control), 1, 5, or 10 μM Lut. To demonstrate the protective effect of Lut against oxidative stress, additional IVM experiments were performed in the absence or presence of 1 mM H_2_O_2_ (Do et al., 2015), depending on the experimental design.

### Parthenogenetic Activation

Metaphase II oocytes were parthenogenetically activated in a 1-mm gab wire chamber (CUY5000P1, Nepagene, Chiba, Japan) with 10 μL of 280 mM mannitol solution containing 0.1 mM MgSO4⋅7H_2_O, 0.1 mM CaCl_2_⋅2H_2_O, 0.5 mM HEPES, and 1 mg/mL polyvinyl alcohol (PVA). Oocytes were activated with a 110-V direct current for 50 μs using an electro cell fusion generator (LF101, Nepagene). Activated oocytes were transferred into *in vitro* culture (IVC) medium (porcine zygote medium-3 containing 4 mg/mL BSA) supplemented with 5 μg/mL cytochalasin B and 2 mM 6-dimethylaminopurine for 4 h at 38.5°C under 5% CO_2_. After 4 h, the oocytes were transferred to IVC medium at 38.5°C under 5% CO_2_. Cleavage and blastocyst formation were evaluated on days 2 and 6, respectively.

### IVF

*In vitro* fertilization was performed in modified *Tris*-buffered medium (mTBM) consisting of 113.1 mM NaCl, 3 mM KCl, 7.5 mM CaCl_2_⋅2H_2_O, and 20 mM *Tris* (crystallized free base; Fisher Scientific, Waltham, MA, United States), 11 mM glucose, 5 mM sodium pyruvate, 2.5 mM caffeine sodium benzoate, and 1 mg/mL BSA. Ejaculated fresh swine semen was washed three times by centrifugation for 3 min at 100*g* and room temperature with Dulbecco’s phosphate-buffered saline (DPBS; Gibco, Grand Island, NY, United States) supplemented with 1 mg/mL BSA, 100 μg/mL penicillin G, and 75 μg/mL streptomycin sulfate. Washed spermatozoa were re-suspended in mTBM for 15 min. Next, 2 μL of diluted spermatozoa was added to 48 μL of mTBM containing 10–15 oocytes, yielding a final concentration of 1.5 × 10^5^ spermatozoa/mL. The oocytes were co-incubated with the spermatozoa for 6 h at 38.5°C under 5% CO_2_. After 6 h, spermatozoa covering the oocytes were stripped via gentle pipetting. Thereafter, the IVF embryos were incubated in IVC medium at 38.5°C under 5% CO_2_ for 6 days. Cleavage and blastocyst formation were evaluated on days 2 and 6, respectively.

### Measurement of Intracellular ROS Levels

Oocytes from each treatment group were incubated for 10 min in DPBS (Welgene, Gyeongsan, South Korea) supplemented with 1 mg/mL PVA (DPBS–PVA) mixed with 10 μM CM-H2DCFDA (Invitrogen, Paisley, United Kingdom). After incubation, the oocytes were washed with DPBS–PVA, and fluorescence was observed under a fluorescence microscope (DMI 4000B; Leica, Wetzlar, Germany). The fluorescence intensities of the oocytes were analyzed using ImageJ software (version 1.47; National Institutes of Health, Bethesda, MD, United States) and normalized to those of the control oocytes.

### Confocal Microscopy of Actin Filaments

Metaphase II oocytes were fixed in 10% neutral buffered formalin solution overnight at 4°C. Fixed oocytes were permeabilized in DPBS (Welgene) containing 0.5% (*v/v*). Triton X-100 for 30 min at room temperature and blocked in blocking solution (DPBS–PVA supplemented with 2 mg/mL BSA) for 1 h at room temperature. The oocytes were stained with 10 μg/mL phalloidin-tetramethylrhodamine B isothiocyanate for 2 h at room temperature. After washing in DPBS–PVA, the oocytes were mounted on glass slides with 1.5 μg/mL 4, 6-diamidino-2-phenylindole (DAPI; Vector Laboratories, Inc., Burlingame, CA, United States) and observed using a laser-scanning confocal fluorescent microscope (LSM700; Zeiss, Oberkochen, Germany).

### Confocal Microscopy of α-Tubulin

After 28 h of IVM, denuded oocytes were fixed in formalin solution for at least 4 h at 38.5°C and permeabilized in DPBS (Welgene) containing 0.5% (*v/v*) Triton X-100 for 1 h at room temperature. Then, the oocytes were blocked in blocking solution for 1 h at room temperature. Next, the oocytes were stained with 1 μg/mL anti-α-tubulin antibody (Invitrogen) overnight at 4°C. The oocytes were washed in DPBS (Welgene) containing 0.05% (*v/v*) Tween 20 and then blocked again under the same conditions. The oocytes were incubated for 1 h at room temperature with a conjugated secondary antibody-Alexa Fluor 488-labeled goat anti-mouse IgG (1:200 in blocking solution) and washed in DPBS (Welgene) containing 0.05% (*v/v*) Tween 20. Oocytes were mounted on glass slides with 1.5 μg/mL DAPI and observed using a laser-scanning confocal fluorescent microscope (LSM700; Zeiss).

### Analysis of Mitochondrial Distribution, Mitochondrial Membrane Potential, and Mitochondrial ROS

Mitochondrial distribution and the mitochondrial membrane potential (MMP) were detected using MitoTracker Deep Red (Invitrogen) and JC-1 (Cayman Chemical, Ann Arbor, MI, United States), with red fluorescence indicating the aggregated form (J-aggregate) and green fluorescence indicating the monomer form (J-monomer). Additionally, mitochondrial ROS (mtROS) were detected using MitoSOX. MII oocytes were fixed in formalin solution for 1 h at 38.5°C and then washed in DPBS–PVA. The oocytes were incubated with 200 nM MitoTracker in DPBS–PVA, JC-1 (1:100), or 10 μM MitoSOX at 38.5°C for 30 min and then washed with DPBS–PVA. Fluorescence signals were detected using a fluorescence microscope (DMi8; Leica). The fluorescence intensities of the oocytes were analyzed using ImageJ software (version 1.47) and normalized to those of the control oocytes.

### Analysis of ER Distribution and Cytoplasmic Calcium Concentration

Metaphase II oocytes were fixed in formalin solution for 1 h. Washed oocytes were then incubated with 1 μM ER Tracker (Invitrogen) or 10 μM Fluo-3 (Invitrogen) dissolved in DMSO plus 0.05% Pluronic F-127 for 30 min. After the oocytes were washed in DPBS–PVA, fluorescence signals were detected using a fluorescence microscope (DMi8; Leica). The fluorescence intensities of the oocytes were analyzed using ImageJ software (version 1.47) and normalized to those of the control oocytes.

### Confocal Microscopy of Cortical Granules

Metaphase II oocytes were fixed in formalin solution at 38.5°C for 3 h and then washed in DPBS–PVA supplemented with 3 mg/mL BSA and 100 mM glycine. The oocytes were incubated in DPBS containing 0.1% (*v/v*) Triton X-100 for 5 min at room temperature and then incubated with 10 μg/mL fluorescein isothiocyanate (FITC)-labeled peanut agglutinin (Vector Laboratories, Inc.) for 30 min. Subsequently, the oocytes were washed in DPBS–PVA supplemented with 3 mg/mL BSA and 0.01% (*v/v*) Triton X-100. The oocytes were mounted with 1.5 μg/mL DAPI and cortical granules (CGs) are observed using a laser-scanning confocal fluorescent microscope (LSM700; Zeiss).

### Terminal Deoxynucleotidyl Transferase-Mediated dUTP-Digoxygenin Nick End-Labeling Assay

A terminal deoxynucleotidyl transferase-mediated dUTP-digoxygenin nick end-labeling (TUNEL) assay was conducted using an *In Situ* Cell Death Detection kit (Roche, Basel, Switzerland). On day 6, blastocysts were fixed in formalin solution for 1 h at room temperature. The fixed blastocysts were incubated in DPBS (Welgene) containing 1% (*v/v*) Triton X-100 for 1 h at room temperature, and stained with fluorescein-conjugated dUTP and terminal deoxynucleotidyl transferase for 1 h at 38.5°C. As a negative control for the TUNEL reaction, a group of blastocysts was incubated in fluorescein-conjugated dUTP in the absence of terminal deoxynucleotidyl transferase. Thereafter, blastocysts were mounted on slides with 1.5 μg/mL DAPI, and DAPI-labeled or TUNEL-positive nuclei were observed under a fluorescence microscope (DMi8; Leica).

### Cdx2 Staining

On day 6, blastocysts were fixed in formalin solution for 1 h at room temperature. The fixed blastocysts were then washed and incubated in DPBS (Welgene) containing 1% (*v/v*) Triton X-100 for 1 h at room temperature, washed in DPBS–PVA, and blocked in DPBS–PVA supplemented with 1 mg/mL BSA (DPBS–PVA–BSA) at 4°C overnight. Next, the blastocysts were blocked with 10% normal goat serum for 45 min and then incubated overnight at 4°C with the primary antibody-mouse monoclonal anti-Cdx2 (undiluted solution; Biogenex Laboratories, Inc., San Ramon, CA, United States). Next, the blastocysts were washed in DPBS–PVA–BSA and incubated for 1 h at room temperature with the Alexa Fluor 488-labeled goat anti-mouse IgG conjugated secondary antibody (1:200 in DPBS–PVA–BSA). Finally, the blastocysts were washed in DPBS–PVA–BSA, and DNA was stained with 1.5 μg/mL DAPI. DAPI-labeled or Cdx2-positive nuclei were observed using a fluorescence microscope (DMi8; Leica).

### Quantitative Real-Time Polymerase Chain Reaction

Poly(A) mRNAs were extracted from 10 MII oocytes or blastocysts using the Dynabeads mRNA Direct Micro kit (Invitrogen, Paisley, United Kingdom). Samples were lysed in 100 μL of lysis/binding buffer at room temperature for 5 min, and 30 μL of Dynabeads oligo (dT)^25^ was added to each sample. The beads were separated from the binding buffer using a Dynal magnetic bar (Invitrogen). Bound poly(A) mRNAs and beads were washed with buffers A and B and then separated by adding 7 μL of *Tris* buffer. Prior to reverse transcription, RNAase contamination was removed with 3 μL of cleansing solution containing genomic DNA (gDNA) Eraser and 5X gDNA Eraser buffer. The resulting poly(A) mRNAs were reverse-transcribed in 10-μL reactions containing Primescript RT Enzyme Mix, 5X Primescript buffer, and RT Primer Mix (Takara, Osaka, Japan). The secondary RNA structure was denatured at room temperature for 5 min to facilitate cDNA production. The reaction was terminated by incubation at 37°C for 15 min and 85°C for 5 s. The resulting cDNA was used as a template for polymerase chain reaction (PCR) amplification. The following PCR conditions were used: 95°C for 20 s and 60°C for 20 s. The Mx3000P QPCR system (Agilent, Santa Clara, CA, United States) and SYBR Premix Ex Taq (Takara Bio, Inc., Shiga, Japan) were used for quantitative real-time (qRT)-PCR. The threshold cycle (Ct) is defined as the fractional cycle number at which the fluorescence passes a fixed threshold above baseline. For the comparative analyses, mRNA expression levels were normalized to that of *H2A* and are expressed as in terms of fold-changes. The sample delta Ct (SΔCT) value was calculated as the difference between the Ct values of *H2A* and the target genes. The relative gene expression levels between the samples and control were determined using the formula 2^–(SΔ*CT*–*C*Δ*CT)*^. The primers used in the current study are listed in Supplementary Table 1.

### Statistical Analyses

**FIGURE 1:**
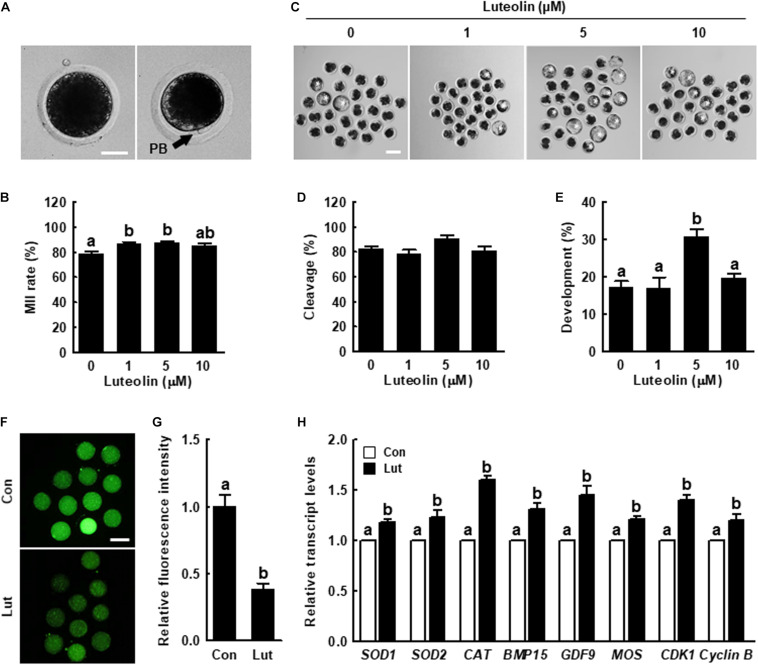
Effects of luteolin (Lut) on meiotic maturation and subsequent embryonic development in porcine embryos after parthenogenetic activation. **(A)** Representative images of porcine oocytes with or without polar bodies. Scale bar = 50 μm. **(B)** Percentages of metaphase II (MII) oocytes after *in vitro* maturation (IVM) in the indicated groups (0 μM, *n* = 182; 1 μM, *n* = 180; 5 μM, *n* = 178; 10 μM, *n* = 182). **(C)** Representative images of blastocyst formation in the indicated groups. Scale bar = 100 μm. Rates of **(D)** cleavage and **(E)** blastocyst formation in the indicated groups (0 μM, *n* = 103; 1 μM, *n* = 103; 5 μM, *n* = 111; 10 μM, *n* = 106). **(F)** Representative fluorescent images and **(G)** relative intensity levels of reactive oxygen species (ROS) in the indicated groups (control [Con], *n* = 28; Lut, *n* = 28). Scale bar = 100 μm. **(H)** Relative expression of oxidative stress- and oocyte competence-related genes in the indicated groups. The data are from at least three independent experiments, and the different superscript letters represent the significant difference (*p* < 0.05).

All experiments were repeated at least three times, and data are presented as the mean and standard error of the mean. The results were compared via one-way analysis of variance, followed by Tukey’s multiple-range tests, unless Student’s *t*-test (Figures 1G,H) was indicated, using PASW Statistics for Windows, Version 18 (SPSS Inc., Chicago, IL, United States). *p*-values less than 0.05 were considered to denote statistical significance.

## Results

### Lut Enhances Porcine Oocyte Quality by Reducing Intracellular ROS Levels

To examine the effect of Lut on porcine oocyte meiotic maturation and subsequent embryonic development, we cultured the COCs in IVM medium supplemented with different concentrations of Lut (0, 1, 5, and 10 μM). The proportion of MII oocytes was significantly higher in the 1 and 5 μM Lut groups than in the other groups (Figures 1A,B and Supplementary Table 2). The blastocyst formation rate after parthenogenetic activation was significantly improved in the 5 μM Lut group (Figures 1C–E and Supplementary Table 3). Based on these results, we used 5 μM Lut for the following experiments.

We measured intracellular ROS levels to determine the antioxidant activity of Lut in oocytes. ROS levels were remarkably decreased in the Lut group compared with the control (Figures 1F,G). The expression levels of oxidative stress (*SOD1*, *SOD2*, and *CAT*)-, oocyte competence (*BMP15* and *GDF9*)-, mitogen-activated protein kinase (MAPK; *MOS*)-, and maturation promoting factor (*CDK1* and *Cyclin B*)-related genes in the Lut group were significantly higher than in the control (Figure 1H). Next, we cultured COCs in IVM medium supplemented with Lut and/or H_2_O_2_ and recorded intracellular ROS levels and the proportion of MII oocytes. The results showed that H_2_O_2_-expose significantly increased intracellular ROS levels, and Lut supplementation reduced this increase to control level (Supplementary Figure 1). Furthermore, the proportion of MII oocytes was lower in H_2_O_2_-exposed oocytes, but this decrease was rescued by Lut supplementation (Supplementary Table 4). These results showed that Lut supplementation during IVM improves meiotic progression by reducing oxidative stress in porcine oocytes.

### Lut Alleviates Oxidative Stress-Induced Actin and Spindle Defects in Porcine Oocytes

**FIGURE 2:**
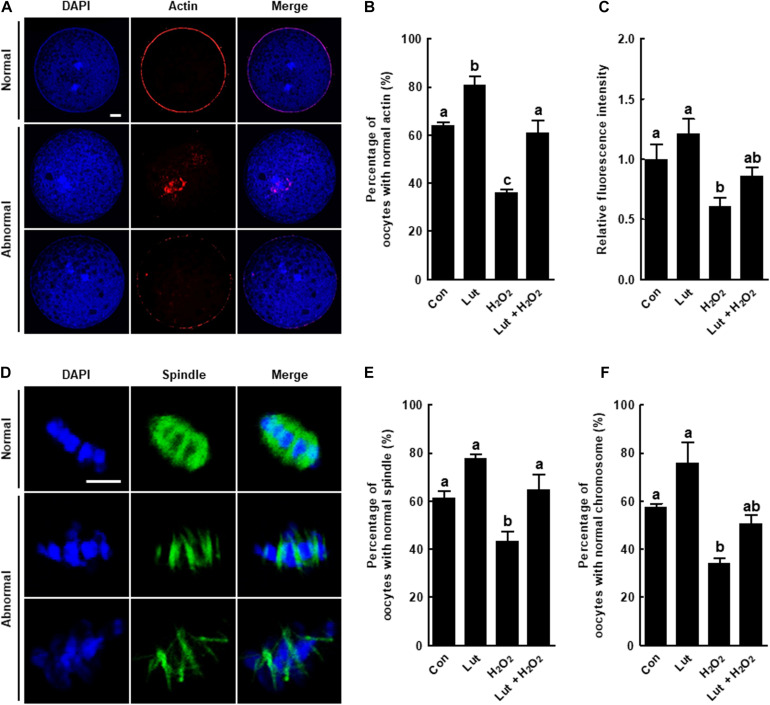
Effects of Lut on actin and spindle defects induced by H_2_O_2_. **(A)** Representative images of normal and abnormal actin distribution in porcine oocytes. Scale bar = 50 μm. **(B)** Percentages of oocytes with normal actin morphology and **(C)** relative intensities of actin signals in the indicated groups (Con, *n* = 25; Lut, *n* = 25; H_2_O_2_, *n* = 25; Lut + H_2_O_2_, *n* = 25). **(D)** Representative images of a normal and abnormal spindle and chromosome structures. Scale bar = 10 μm. Percentages of oocytes with normal **(E)** spindle and **(F)** chromosome morphology in the indicated groups (Con, *n* = 32; Lut, *n* = 29; H_2_O_2_, *n* = 37; Lut + H_2_O_2_, *n* = 29). The data are from at least three independent experiments, and the different superscript letters represent the significant difference (*p* < 0.05).

We examined the actin and spindle morphology in Lut and/or H_2_O_2_-treated oocytes. The percentage of oocytes with normal actin morphology, with the actin accumulating uniformly on the plasma membrane, was higher in the Lut group than in the control. Actin filament signals from the H_2_O_2_-exposed oocytes were indicative of more abnormal morphology, such as a much weaker or discontinuous distribution, but these were restored by Lut supplementation (Figures 2A,B). The quantification of actin fluorescence signals confirmed the observations. The fluorescent intensity of actin was reduced in the oocytes exposed to H_2_O_2_, but the decrease was attenuated by Lut supplementation (Figure 2C). Moreover, the proportion of oocytes with well-organized spindle and chromosome structures was significantly lower in the H_2_O_2_ group than in the other groups, with the decrease restored by Lut supplementation (Figures 2D–F). In short, Lut supplementation during IVM improves cytoskeleton dynamics-mediated nuclear maturation by reducing oxidative stress.

### Lut Recovers the Mitochondrial Content and MMP in Porcine Oocytes by Regulating the mtROS Level

**FIGURE 3:**
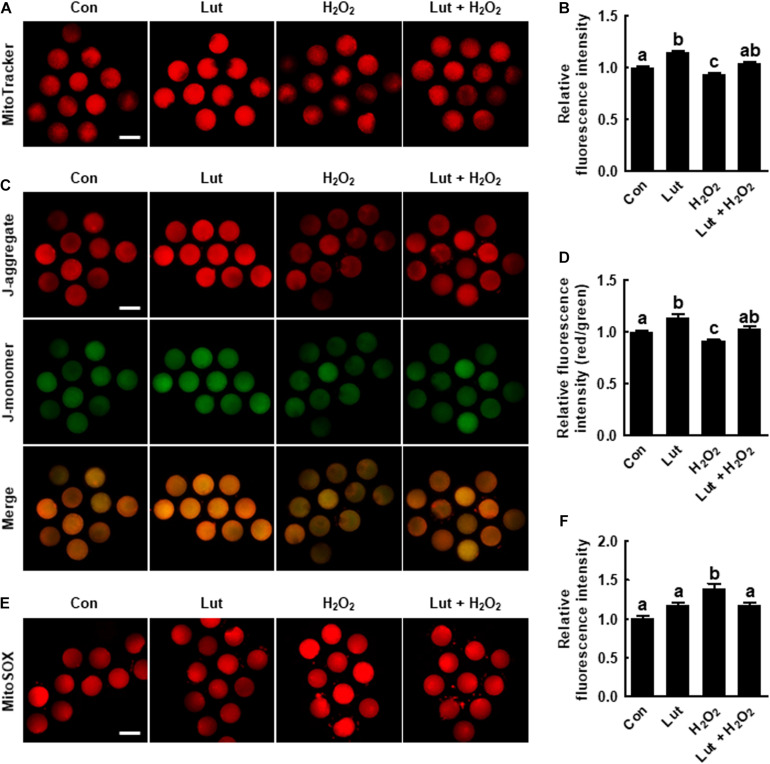
Effects of Lut on H_2_O_2_-induced mitochondrial dysfunction and superoxide production. **(A)** Representative images of porcine oocytes stained with MitoTracker and **(B)** relative fluorescence intensities in the indicated groups (Con, *n* = 35; Lut, *n* = 35; H_2_O_2_, *n* = 35; Lut + H_2_O_2_, *n* = 35). Scale bar = 100 μm. **(C)** Representative images of porcine oocytes stained with JC-1 and **(D)** the ratios of red to green fluorescence intensity in the indicated groups (Con, *n* = 35; Lut, *n* = 35; H_2_O_2_, *n* = 35; Lut + H_2_O_2_, *n* = 35). Scale bar = 100 μm. **(E)** Representative images of porcine oocytes stained with MitoSOX and **(F)** relative fluorescence intensities in the indicated groups. Scale bar = 100 μm. (Con, *n* = 34; Lut, *n* = 34; H_2_O_2_, *n* = 34; Lut + H_2_O_2_, *n* = 34). The data are from at least three independent experiments, and the different superscript letters represent the significant difference (*p* < 0.05).

We explored the mitochondrial content, MMP, and mtROS level in Lut- and/or H_2_O_2_-treated oocytes. MitoTracker intensity levels were higher in Lut-treated oocytes and lower in H_2_O_2_-treated oocytes compared with the control (Figures 3A,B). Consistently, the ratio of fluorescence intensity of the J-aggregate (high membrane potential) to that of the J-monomer (low membrane potential), which is an index of the MMP, was significantly increased in Lut-treated oocytes and decreased in H_2_O_2_-exposed oocytes compared with the control (Figures 3C,D). Lut restored the reduced mitochondrial content and MMP. Conversely, the fluorescence intensity of MitoSOX was markedly higher in the H_2_O_2_-exposed oocytes than in the control and Lut-treated oocytes, but this increase was reduced upon Lut treatment (Figures 3E,F). These results indicate that Lut supplementation during IVM improves mitochondrial function by alleviating oxidative stress.

### Lut Restores Oxidative Stress-Induced ER Function Defects in Porcine Oocytes

**FIGURE 4:**
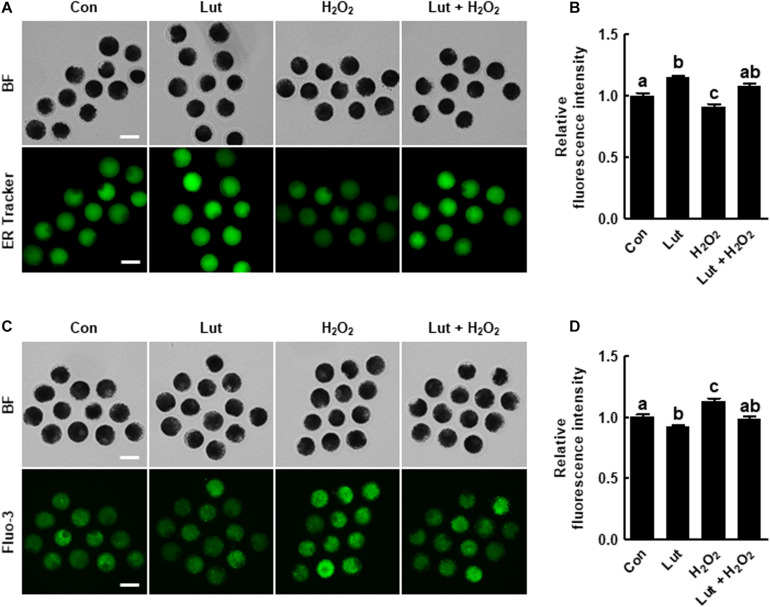
Effects of Lut on endoplasmic reticulum (ER) defects induced by H_2_O_2_. **(A)** Representative images of porcine oocytes stained with ER Tracker and **(B)** relative fluorescence intensities in the indicated groups (Con, *n* = 40; Lut, *n* = 40; H_2_O_2_, *n* = 40; Lut + H_2_O_2_, *n* = 40). Scale bar = 100 μm. **(C)** Representative fluorescent images and **(D)** relative fluorescence intensities representing intracellular calcium levels in oocytes stained with Fluo-3 in the indicated groups (Con, *n* = 38; Lut, *n* = 38; H_2_O_2_, *n* = 38; Lut + H_2_O_2_, *n* = 38). Scale bar = 100 μm. The data are from at least three independent experiments, and the different superscript letters represent the significant difference (*p* < 0.05).

To investigate the effects of Lut on the ER contents and function in porcine oocytes, we evaluated the ER content and intracellular calcium levels in Lut- and/or H_2_O_2_-treated oocytes. The ER Tracker fluorescence level was significantly higher in the Lut group and lower in the H_2_O_2_ group compared with the control. Lut recovered the reduction in fluorescence intensity caused by H_2_O_2_ exposure (Figures 4A,B). Additionally, the intensity level of Fluo-3 was remarkably lower in Lut-treated oocytes and higher in H_2_O_2_-exposed oocytes compared with the control, but the increased fluorescence intensity due to H_2_O_2_ exposure was altered to the control level by Lut supplementation (Figures 4C,D). These results suggest that Lut supplementation during IVM maintains ER function in the face of oxidative stress.

### Lut Recovers the Distribution of CGs in H_2_O_2_-Exposed Porcine Oocytes

**FIGURE 5:**
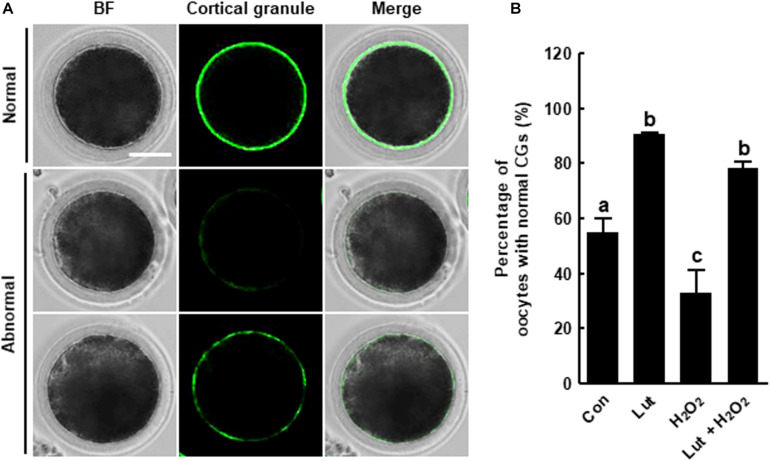
Effects of Lut on H_2_O_2_-induced abnormal distribution of cortical granules (CGs). **(A)** Representative images of the normal and abnormal morphologies of CGs in porcine oocytes. Scale bar = 50 μm. **(B)** Percentages of oocytes with a normal CG distribution in the indicated groups (Con, *n* = 44; Lut, *n* = 43; H_2_O_2_, *n* = 37; Lut + H_2_O_2_, *n* = 41). The data are from at least three independent experiments, and the different superscript letters represent the significant difference (*p* < 0.05).

We investigated the distribution of CGs in Lut- and/or H_2_O_2_-treated oocytes using FITC-labeled peanut agglutinin staining. The percentage of oocytes with a normal CG distribution, which indicates localization in the cortex with a continuous and strong signal, was higher in the Lut group and lower in the H_2_O_2_ group compared with the control. H_2_O_2_-exposed oocytes exhibited much weaker, discontinuous, or almost completely absent subcortical localization. Interestingly, Lut supplementation remarkably restored these defects, indicating that Lut supplementation during IVM improves the dynamics of CGs (Figure 5).

### Lut Supplementation During IVM Improves the Developmental Competence of Porcine IVF Embryos

**FIGURE 6:**
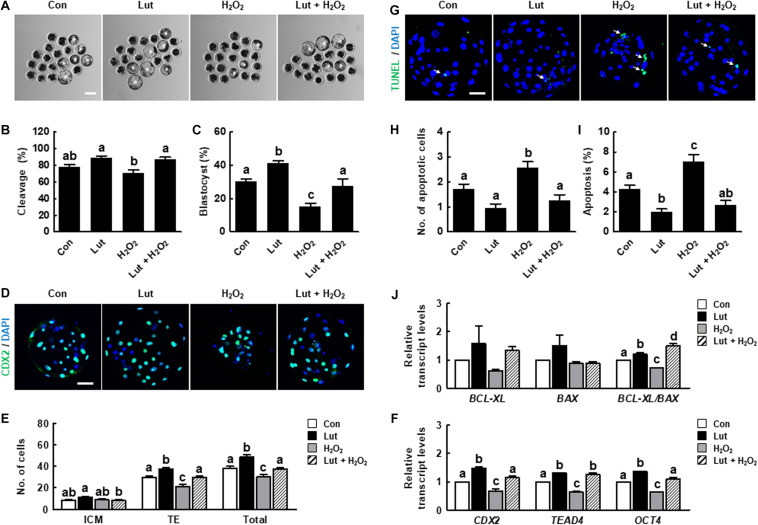
Effects of Lut during IVM on the developmental competence of *in vitro* fertilization (IVF) embryos. **(A)** Representative images of blastocyst formation at 6 days after IVF in the indicated groups. Scale bar = 100 μm. Rates of **(B)** cleavage and **(C)** blastocyst formation in the indicated groups (Con, *n* = 144; Lut, *n* = 145; H_2_O_2_, *n* = 134; Lut + H_2_O_2_, *n* = 133). **(D)** Representative images of Cdx2- and DAPI-stained blastocysts in the indicated groups. Scale bar = 50 μm. **(E)** Number of inner cell mass (ICM), trophectoderm (TE), and total cells in the indicated groups (Con, *n* = 20; Lut, *n* = 20; H_2_O_2_, *n* = 20; Lut + H_2_O_2_, *n* = 20). **(F)** Relative expression of ICM/TE differentiation-related genes in the indicated groups. **(G)** Representative images of TUNEL- and DAPI-stained blastocysts in the indicated groups. Scale bar = 50 μm. **(H)** Number of apoptotic cells and **(I)** the apoptosis rate in the indicated groups (Con, *n* = 20; Lut, *n* = 20; H_2_O_2_, *n* = 20; Lut + H_2_O_2_, *n* = 20). **(J)** Relative expression of apoptosis-related genes and the ratio of *BCL-XL*/*BAX* levels in the indicated groups. The data are from at least three independent experiments, and the different superscript letters represent the significant difference (*p* < 0.05).

We assessed developmental competence in Lut- and/or H_2_O_2_-treated oocytes following IVF. As shown in Figures 6A–C and Supplementary Table 5, the cleavage rate in the Lut and H_2_O_2_ co-treatment group was significantly higher than that in the H_2_O_2_ group. The blastocyst formation rate was significantly higher in the Lut group and lower in the H_2_O_2_ group compared with the control. Lut supplementation recovered the reduced blastocyst formation rate to the control level. Next, we characterized the blastocyst quality using Cdx2 and TUNEL analysis. The numbers of total blastocyst cells and trophectoderm (TE) cells increased in the Lut group and decreased in the H_2_O_2_ group, with Lut recovering the reductions in cell number (Figures 6D,E and Supplementary Table 6). The expression levels of inner cell mass (ICM)/TE differentiation-related genes were significantly increased in the Lut group and decreased in the H_2_O_2_ group. Lut supplementation restored the reductions due to H_2_O_2_ (Figure 6F). Moreover, the apoptotic cell number in the H_2_O_2_ group was remarkably higher than those in the other groups (Figures 6G–I and Supplementary Table 7). The rate of cell apoptosis was higher in the H_2_O_2_ group and lower in the Lut group, with Lut supplementation alleviating the increase in apoptosis. The ratio of *BCL-XL* to *BAX* expression was significantly higher in the Lut group and lower in the H_2_O_2_ group compared with the control. Lut supplementation recovered the reduction due to H_2_O_2_ (Figure 6J). Collectively, these results showed that Lut treatment during IVM can improve the developmental competence of porcine embryos.

## Discussion

In the present study, we investigated the effects of Lut on the IVM and subsequent developmental competence of porcine oocytes. ROS are byproducts of metabolism, and excessive ROS levels cause oxidative injury to cells that may result in DNA damage, lipid peroxidation, mitochondrial defects, and cell death (Kang et al., 2021). In oocytes, a disrupted ROS balance reduces developmental competence compared with *in vivo* oocytes, as shown by decreased rates of polar body extrusion, cleavage, and blastocyst formation in mammalian oocytes (Rizos et al., 2002; Qian et al., 2016). Thus, it is necessary to improve developmental competence by investigating the regulation of oxidative stress and the mechanisms related to the effects of various antioxidants on oocyte maturation.

Flavonoids are polyphenols that protect plant cells against microorganisms, insects, and UV irradiation. Previous studies have shown that flavonoids such as kaempferol and quercetin exert antioxidant effects on mammalian oocytes (Cao et al., 2020; Zhao et al., 2020). Lut, a flavone in the flavonoid group, also exhibits antioxidant activity. Lut can stabilize the radical group by donating hydrogen/electron of C2–C3 double bond to radical and by blocking Fenton reaction using oxo group at C4 that binds transitional metal ions including iron and copper. These two structural features of Lut inhibit pro-oxidant enzymes such as xanthine oxidase, and induce antioxidant enzymes (Gendrisch et al., 2021). In addition, Lut not only has its own antioxidant activity, but also interacts with other antioxidants such as vitamins and cellular redox system (Gendrisch et al., 2021), indicating that Lut can synergistically enhance its antioxidant properties. Furthermore, some flavonoids including quercetin, genistein, and catechin induce DNA damage by pro-oxidative effect resulting in mutagenic and carcinogenic activity (Kawanishi et al., 2005; Gendrisch et al., 2021), but Lut has no report regarding these effects, suggesting that Lut can be expected as a comparatively safe antioxidant (Yamashita et al., 1999). In the present study, we demonstrated for the first time that Lut supplementation improves oocyte maturation and subsequent embryonic development. Moreover, Lut supplementation decreased intracellular ROS levels and increased the expression levels of antioxidant-related genes. These results indicate that Lut exerts positive effects on IVM porcine oocytes by reducing oxidative stress.

The MAPK signaling pathway is crucial for the female reproduction process including the embryo development and meiotic maturation (Chen et al., 2020). The extracellular signal-regulated kinase-1/2 (ERK1/2) is a member of MAPK signaling pathway and is an important signaling molecule, which functions microfilament or microtubule dynamics, during oocyte maturation (Hatch and Capco, 2001). In addition, phosphorylation of ERK1/2 (p-ERK1/2) at MII stage is considered to parameter of cytoplasmic maturation that affect cleavage and the blastocyst formation rate after fertilization (Inoue et al., 1995). However, MAPK signaling pathway disorder is one of pathways that cause oxidative damage (Yao et al., 2019). Previous studies reported that Lut upregulated the expression of p-ERK1/2 and MAPK, suggesting Lut alleviate oxidative stress by activating the MAPK signaling pathway (Wu et al., 2015; Wang et al., 2020). In this study, Lut treatment increased the developmental competence of PA and IVF embryos. Moreover, Lut treatment increased expression of oocyte competence-, MAPK, and maturation promoting factor-related genes. These results indicate Lut improves oocyte meiotic progression and subsequent embryonic development against oxidative stress.

A proper cytoskeletal system is crucial for meiotic maturation, as the cytoskeleton controls chromosome condensation and segregation, subsequent meiosis, fertilization, and further cell cleavage (Brunet and Maro, 2005). Within the cytoskeleton, microfilaments are involved in chromosome migration, cortical spindle anchorage, and first polar body emission, whereas microtubules facilitate chromosome movement by organizing spindles (Sun and Schatten, 2006; Duan and Sun, 2019). However, previous studies showed that oxidative stress impaired cytoskeletal dynamics, with the disrupted microfilament or microtubule dynamics resulting in defective polar body extrusion (Zhang et al., 2018). Actin is a microfilament subunit, and tubulin is a microtubule subunit. We investigated the dynamics of these subunits. Consistent with previous study, our results showed that oxidative stress caused by H_2_O_2_ exposure disrupted microfilament or microtubule dynamics resulting in poor proportion of MII oocytes. Interestingly, Lut treatment completely or partially rescued these defects. Lut treatment significantly recovered the decreased proportion of MII oocytes and the disruption of actin and spindle structures caused by H_2_O_2_ exposure, although actin amounts and chromosome structure were not rescued to the control level. Thus, we suggest that Lut enhances nuclear maturation by normalizing cytoskeletal dynamics against oxidative stress.

Oocyte maturation includes a series of complex events, such as protein synthesis and the transcription of cytoplasmic RNA, which require energy. The ER functions in protein folding and degradation, and mitochondria supply energy for protein synthesis (Dumollard et al., 2007). The ER also acts as the major storage area for calcium ions (Ca^2+^), thus regulating intracellular Ca^2+^ homeostasis. Ca^2+^ is one of the major signal molecules that regulate oocyte physiology, including cell cycle resumption (Tiwari et al., 2017). Abnormally high concentrations of Ca^2+^ in the cytoplasm result in cell cycle arrest, disruption of fertilization ability, and apoptosis (Tiwari et al., 2015; Wang et al., 2017). Oxidative stress causes Ca^2+^ influx into the cytoplasm from the ER and subsequently increases the mitochondrial Ca^2+^ concentration (Ermak and Davies, 2002). Mitochondrial Ca^2+^ overload is a critical sensitizing signal in the apoptosis pathway that causes embryonic developmental arrest and death (Marchi et al., 2018; Kim et al., 2020). Moreover, severe oxidative stress triggers a decrease in cellular mitochondrial content through the suppression of mitochondrial biogenesis as well as in mitochondrial function (Bouchez and Devin, 2019; Jeong et al., 2020). Our results showed that Lut protects the ER and mitochondrial function against oxidative stress, with Lut supporting proper cytoplasmic maturation through regulation of the ER and mitochondrial system.

Finally, we investigated the fertilization ability and developmental competence of Lut-treated oocytes after IVF. CGs are organelles located in the subcortical region of oocytes, and the distribution of CGs is regarded as a marker of oocyte maturation. Additionally, as CGs are related to the blocking of polyspermy, proper CG dynamics are crucial for successful fertilization (Burkart et al., 2012). In the present study, Lut normalized the distribution of CGs and enhanced developmental competence in the face of oxidative stress, as shown in the cleavage and blastocyst formation rate, cell number, and apoptotic pattern, suggesting that Lut supplementation during IVM has positive effects on the developmental competence of IVF embryos.

**FIGURE 7:**
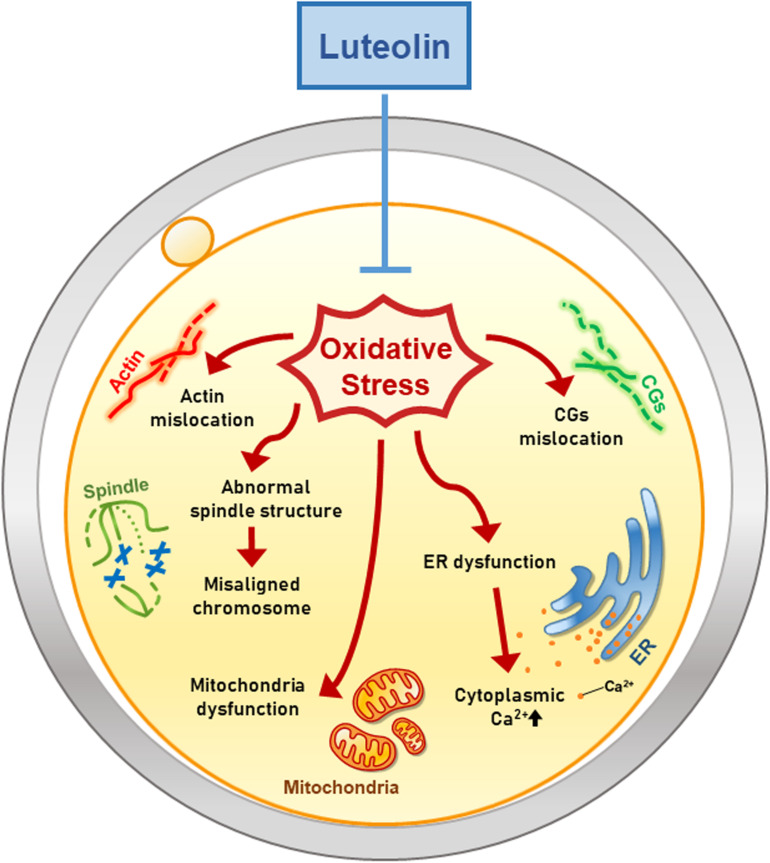
Hypothetical model of the antioxidant effect of Lut on porcine oocyte maturation. Lut improves the quality of porcine oocytes by alleviating oxidative damage. Especially, Lut recovers disrupted organelle dynamics including mislocation of actin, spindle, chromosome and CGs, and dysfunction of mitochondria and ER. These findings suggest that Lut improves porcine meiotic progression and subsequent embryonic development by protecting various organelle dynamics against oxidative stress.

This is the first study to demonstrate the effects of Lut on mammalian oocytes (Figure 7). Moreover, we especially focused on the dynamics of cell organelles during IVM under oxidative stress and subsequently determined the antioxidant activities of Lut. Our study suggests that Lut improves the quality of porcine oocytes and subsequent embryonic development following IVF by alleviating oxidative damage to organelles. These findings help raise awareness of the beneficial effects of Lut on IVM and elucidate how Lut supports proper oocyte maturation under oxidative stress.

## Data Availability Statement

The original contributions presented in the study are included in the article/Supplementary Material, further inquiries can be directed to the corresponding authors.

## Author Contributions

S-HP and P-SJ designed study, performed experiments, analyzed data, and wrote the manuscript. YJ, H-GK, MK, SL, and B-SS performed experiments and analyzed data. S-UK acquired financial and discussed study. S-KC and B-WS designed study, supervised the study, and wrote the manuscript. All authors have read and agreed to the published final version of this manuscript.

## Conflict of Interest

The authors declare that the research was conducted in the absence of any commercial or financial relationships that could be construed as a potential conflict of interest.
