# Supplementary material for: Luteolin Orchestrates Porcine Oocyte Meiotic Progression by Maintaining Organelle Dynamics Under Oxidative Stress
Source: Front Cell Dev Biol. 2021 Jun 15;9:689826. doi: 10.3389/fcell.2021.689826 (PMC8239245; doi:10.3389/fcell.2021.689826)
Supplement: Supplementary file 1 [file Table_1.DOCX]

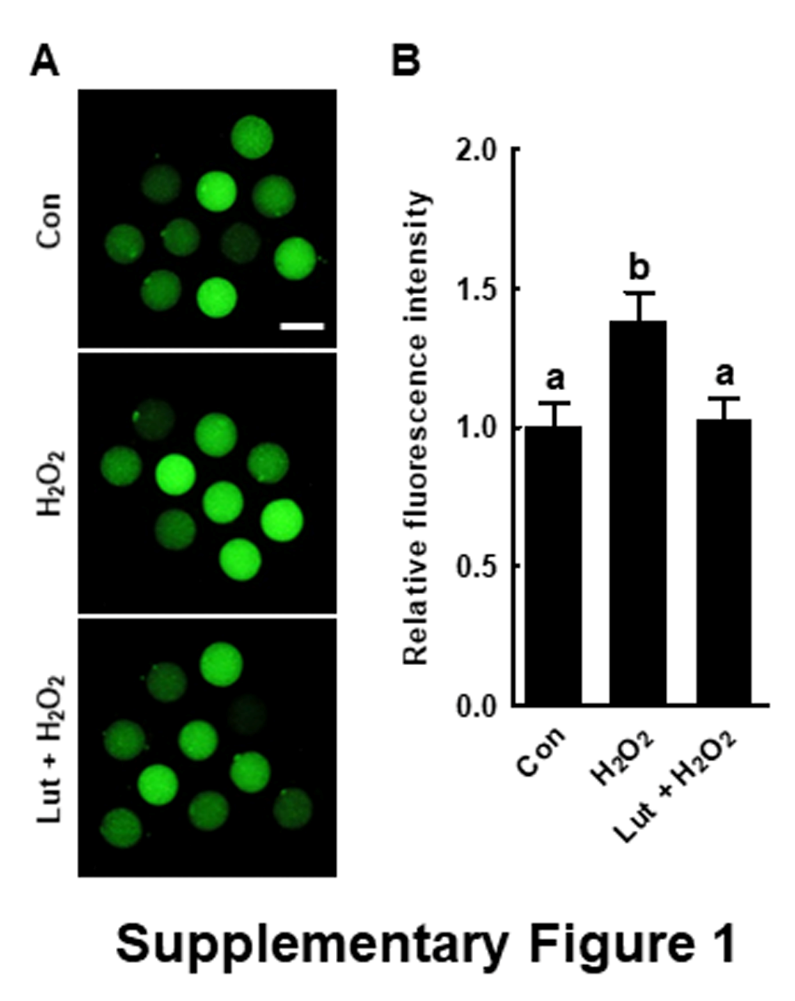


**Supplementary Figure 1.** Effects of Lut on H_2_O_2_-induced intracellular ROS production. **(A)** Representative fluorescent images and **(B)** relative intensity levels of ROS in the indicated groups (Con, *n* = 29; H_2_O_2_, *n* = 29; Lut + H_2_O_2_, *n* = 29). Scale bar = 100 μm. The data are from at least three independent experiments, and the different superscript letters represent the significant difference (*p* < 0.05).

Supplementary Table 1. Primer sequences for Quantitative real-time polymerase chain reaction

| Gene | Primer sequence (5’-3’) | Gene accession no. | Length (bp) |
| --- | --- | --- | --- |
| *H2A* | F: AGTTTCCTGTGGGTCGAGTG | XM_021083382.1 | 162 |
|  | R: TGCGAGTCTTCTTGTTGTC |  |  |
| *BMP15* | F: TGGTGAGGCCATTGGTTAAT | NM_001005155.2 | 156 |
|  | R: AGAGGTGGAAGGGAGCTAGG |  |  |
| *GDF9* | F: AACACTGTCCGGCTCTTCAC | NM_001001909.1 | 202 |
|  | R: CCAGGCTGCACTCACATTTA |  |  |
| *MOS* | F: ATCATCATGGAGTTCGGCGG | NM_001113219.1 | 113 |
|  | R: TCCAAGTTCAGTTGCTCCCC |  |  |
| *CDK1* | F: CAGCTCGCTACTCAACTCCA | NM_001159304.2 | 136 |
|  | R: GGAGTGCCCAAAGCTCTGAA |  |  |
| *Cyclin B* | F: TGTGTGCCCAAGAAGATGCT | NM_001170768.1 | 189 |
|  | R: AGGGCGACCCAGACAAAAAT |  |  |
| *SOD1* | F: GGTGGGCCAAAGGATCAAGA | NM_001190422.1 | 80 |
|  | R: TACACAGTGGCCACACCATC |  |  |
| *SOD2* | F: GGTGGAGGCCACATCAATCA | NM_214127.2 | 220 |
|  | R: AACAAGCGGCAATCTGCAAG |  |  |
| *CAT* | F: TGTACCCGCTATTCTGGGGA | NM_214301.2 | 119 |
|  | R: TCACACAGGCGTTTCCTCTC |  |  |
| *CDX2* | F: GGCAGCCAAGTGAAAACCAG | NM_001278769 | 119 |
|  | R: GCCTTTCTCCGAATGGTGAT |  |  |
| *TEAD4* | F: GATGTACGGTCGGAATGAG | NM_001142666 | 145 |
|  | R: TGCCTGATCCTTTAGCTTGG |  |  |
| *OCT4* | F: AGTGAGAGGCAACCTGGAGA | NM_001113060.1 | 151 |
|  | R: ACTGCTTGATCGTTTGCCCT |  |  |
| *BCL-XL* | F: AGGGCATTCAGTGACCTGAC | NM_214285 | 242 |
|  | R: TGGATCCAAGGCTCTAGGTG |  |  |
| *BAX* | F: AAGCGCATTGGAGATGAACT | XM_003127290 | 251 |
|  | R: CGATCTCGAAGGAAGTCCAG |  |  |

Supplementary Table 2. Effect of luteolin (Lut) concentration on nuclear maturation of porcine oocytes

| Luteolin (μM) | No. of oocytes examined | Immature (%) | Degenerate (%) | Metaphase II (%) |
| --- | --- | --- | --- | --- |
| 0 | 182 | 25 (13.9±2.8) | 13 (7.3±2.5) | 144 (78.9±1.7)^a^ |
| 1 | 180 | 18 (10.1±1.6) | 6 (3.4±1.6) | 156 (86.5±1.5)^b^ |
| 5 | 178 | 14 (7.9±1.3) | 9 (5.0±0.4) | 155 (87.1±1.6)^b^ |
| 10 | 182 | 19 (10.3±1.5) | 9 (4.7±1.4) | 154 (84.9±1.2)^a,b^ |

Data are the mean ± SEM, and values with different superscript letter within a column differ significantly (*p* < 0.05).

Supplementary Table 3. Effect of Lut during *in vitro* maturation (IVM) on the subsequent parthenogenetic activation embryonic development in pig

| Luteolin (μM) | No. of embryos examined | Cleavage (%) | Blastocyst (%) |
| --- | --- | --- | --- |
| 0 | 103 | 78 (75.1±7.5) | 18 (17.1±1.7)^a^ |
| 1 | 103 | 77 (75.2±6.1) | 18 (17.0±2.9)^a^ |
| 5 | 111 | 85 (85.5±4.7) | 34 (30.8±1.8)^b^ |
| 10 | 106 | 75 (70±6.2) | 21 (19.6±1.2)^a^ |

Data are the mean ± SEM, and values with different superscript letter within a column differ significantly (*p* < 0.05).

Supplementary Table 4. Effect of Lut and/or H_2_O_2_ treatment on nuclear maturation of porcine oocyte

| Groups | No. of oocytes examined | Immature (%) | Degenerate (%) | Metaphase II (%) |
| --- | --- | --- | --- | --- |
| Con | 177 | 44 (25±3.2)^a,b,c^ | 4 (2.2±1.6) | 129 (72.8±1.7)^a^ |
| Lut | 182 | 23 (12.9±2.5)^b^ | 7 (3.6±1.7) | 152 (83.4±1.2)^b^ |
| H_2_O_2_ | 181 | 65 (36.2±4.1)^c^ | 10 (5.3±2.3) | 106 (58.6±3.5)^c^ |
| Lut + H_2_O_2_ | 181 | 41 (22.7±0.8)^a,b^ | 8 (4.4±1.3) | 132 (72.8±1.1)^a^ |

Data are the mean ± SEM, and values with different superscript letter within a column differ significantly (*p* < 0.05).

Supplementary Table 5. Effect of Lut and/or H_2_O_2_ treatment during IVM on *in vitro* fertilization (IVF) embryonic development in pig

| Groups | No. of embryos examined | Cleavage (%) | Blastocyst (%) |
| --- | --- | --- | --- |
| Con | 144 | 112 (77.9±2.8)^a,b^ | 43 (30.0±1.6)^a^ |
| Lut | 145 | 129 (88.6±2.7)^a^ | 60 (41.1±1.6)^b^ |
| H_2_O_2_ | 134 | 95 (70.0±4.6)^b^ | 21 (14.9±2.3)^c^ |
| Lut + H_2_O_2_ | 133 | 117 (87.3±2.4)^a^ | 38 (27.5±4.3)^a^ |

Data are the mean ± SEM, and values with different superscript letter within a column differ significantly (*p* < 0.05).

Supplementary Table 6. Effect of Lut and/or H_2_O_2_ treatment during IVM on inner cell mass (ICM), trophectoderm (TE) and total cell number in porcine IVF blastocysts

| Groups | No. of blastocysts  examined | No. of nuclei | | |
| --- | --- | --- | --- | --- |
|  |  | ICM | TE | Total |
| Con | 20 | 8.6±0.6^a,b^ | 29.8±1.5^a^ | 38.4±1.6^a^ |
| Lut | 20 | 11.1±0.9^a^ | 37.5±1.7^b^ | 48.6±2.2^b^ |
| H_2_O_2_ | 20 | 8.9±1.0^a,b^ | 21.4±1.5^c^ | 30.3±1.9^c^ |
| Lut + H_2_O_2_ | 20 | 8.2±0.6^b^ | 29.4±1.4^a^ | 37.6±1.3^a^ |

Data are the mean ± SEM, and values with different superscript letter within a column differ significantly (*p* < 0.05).

Supplementary Table 7. Effect of Lut and/or H_2_O_2_ treatment during IVM on apoptosis in porcine IVF blastocysts

| Groups | No. of blastocysts examined | No. of apoptotic cells | Apoptosis (%) |
| --- | --- | --- | --- |
| Con | 20 | 1.7±0.2^a^ | 4.3±0.4^a^ |
| Lut | 20 | 1.0±0.2^a^ | 2.3±0.3^b^ |
| H_2_O_2_ | 20 | 2.6±0.3^b^ | 7.0±0.7^c^ |
| Lut + H_2_O_2_ | 20 | 1.3±0.2^a^ | 2.7±0.5^a,b^ |

Data are the mean ± SEM, and values with different superscript letter within a column differ significantly (*p* < 0.05).
